# DGK and DZHK position paper on genome editing: basic science applications and future perspective

**DOI:** 10.1007/s00395-020-00839-3

**Published:** 2021-01-15

**Authors:** Ralf P. Brandes, Anne Dueck, Stefan Engelhardt, Manuel Kaulich, Christian Kupatt, Maria Teresa De Angelis, Matthias S. Leisegang, Ferdinand le Noble, Alessandra Moretti, Oliver J. Müller, Boris V. Skryabin, Thomas Thum, Wolfgang Wurst

**Affiliations:** 1 DZHK-German Center for Cardiovascular Research, Berlin, Germany; 2 Institute for Cardiovascular Physiology, Goethe University, Frankfurt am Main, Germany; 3 Institut Für Pharmakologie und Toxikologie, Technische Universität München, Munich, Germany; 4 Institute of Developmental Genetics, Helmholtz Zentrum München Germany, Technische Universität München-Weihenstephan, Neuherberg, Munich, Germany; 5 Institute of Biochemistry II, Medical Faculty, Goethe University Frankfurt, University Hospital, Frankfurt, Germany; 6 First Medical Department (Cardiology), Klinikum rechts der Isar, Technische Universität München, München, Germany; 7 Department of Cell and Developmental Biology, ZOO-2 and ITG, Karlsruhe Institute of Technology (KIT), Karlsruhe, Germany; 8 Department of Internal Medicine III, University of Kiel, Arnold-Heller-Str. 3, 24105 Kiel, Germany; 9 Medical Faculty, Core Facility Transgenic Animal and Genetic Engineering Models (TRAM), University of Muenster, Muenster, Germany; 10 Institute of Molecular and Translational Therapeutic Strategies, Hannover Medical School, Hannover, Germany; 11 Fraunhofer Institute for Toxicology and Experimental Medicine, Fraunhofer Institute, Hannover, Germany; 12 DZNE, German Center for Neurodegenerative Diseases, Site Munich, Munich, Germany; 13 Munich Cluster for Systems Neurology (SyNergy), Munich, Germany

**Keywords:** Animal models, CRISPR/Cas, Genome editing, Animal models

## Abstract

For a long time, gene editing had been a scientific concept, which was limited to a few applications. With recent developments, following the discovery of TALEN zinc-finger endonucleases and in particular the CRISPR/Cas system, gene editing has become a technique applicable in most laboratories. The current gain- and loss-of function models in basic science are revolutionary as they allow unbiased screens of unprecedented depth and complexity and rapid development of transgenic animals. Modifications of CRISPR/Cas have been developed to precisely interrogate epigenetic regulation or to visualize DNA complexes. Moreover, gene editing as a clinical treatment option is rapidly developing with first trials on the way. This article reviews the most recent progress in the field, covering expert opinions gathered during joint conferences on genome editing of the German Cardiac Society (DGK) and the German Center for Cardiovascular Research (DZHK). Particularly focusing on the translational aspect and the combination of cellular and animal applications, the authors aim to provide direction for the development of the field and the most frequent applications with their problems.

## Introduction

It is estimated that approximately 5% of newborns will suffer from a genetic disorder. This suggests that almost 400 million people worldwide are affected by genetic diseases [157], and tailored drugs and/or genetic approaches are lacking for most of them. Gene therapy and especially gene editing are the most rational and mechanistic approaches to treat genetic diseases. Gene editing therefore aims on correcting mutations in a gene, to delete or replace parts of a gene or to alter gene expression. Since the discovery of the genetic code, gene editing has, however, been more a theoretical possibility than practical reality. Limited to a few applications and utilizing imperfect tools, like retrovirus, gene editing, for a long time, did not keep its promises, but this has changed within recent years [78].

In addition to the clinical benefit of gene editing, the technology is also important for the production of transgenic crops and animals. In basic and translational sciences, gene editing also allows specific modifications of genes and manipulation of gene expression in practically all life forms, ranging from cells and organs to living animals. Contrary to transient gain or loss-of-function approaches, like RNAi or plasmid-based overexpression, the effects of gene editing can be permanent, and are therefore less prone to artefacts. The technology, however, also has limitations, like the need of shuttle systems and potential off-target effects.

Within recent years, a series of discoveries has revolutionized the toolset to edit the cellular genome. Programmable, sequence-specific DNA endonucleases now allow for precise genome editing in cultured cells and in vivo, and improvements and modifications in particular in the “clustered regularly interspaced short palindromic repeats” (CRISPR)/Cas9 technology occur at breathtaking speed. In vivo genome editing now becomes possible, and simultaneously, the novel CRISPR/Cas9 applications for basic science allow experiments, simply unimaginable just 10 years ago [154]. This development coincides with profound improvements in bioinformatics and vector development, so that cell targeting becomes more efficient and side-effects can be better predicted, minimized, and controlled.

This position paper, writing on behalf of the commission for experimental cardiology of the German Cardiac Society (DGK) and the German Center for Cardiovascular Research (DZHK), will summarize the different available options of gene editing approaches. It aims at basic and translational scientists, and covers the recent advances in gene editing technologies and their application in unbiased screens and animal studies. Moreover, a perspective for the application of gene editing in cardiovascular medicine will be provided. Finally, the limitations and ethical considerations of gene editing will be addressed and an outlook provided on the considerations for human genome editing and future developments.

## The general approach of gene editing

The traditional approaches of gene editing were restricted to cells and usually based on the integration of linear DNA stretches into the genome, which were provided through transfection or microinjection into the cell. This integration was either targeted, like the technique used for stem cell modification during knockout mouse generation or random [78]. Both techniques heavily relied on antibiotic-based selection, as integration was a rare event, and thus, an antibiotic resistance gene was integrated into the genome, too. Higher integration rates were achieved with retro-viral approaches, but site-specific targeting was limited by these approaches.

The discovery of programmable, sequence-specific nucleases was a paradigm shift for gene editing. Site-specific modifications of the genetic code at fairly high efficiency became possible, which facilitated the generation of designed cellular and animal models. This new era started with the development of sequence-specific nucleases such as meganucleases, zinc-finger nucleases, and TALENs. Just a few years ago, the bacterial CRISPR/Cas9 system was the most important addition to this toolbox and rapidly became the preferred gene editing method based on its simplicity, efficiency, and universality [35]. With these new genetic tools, double-strand breaks can be precisely introduced into the genome. Depending on the subsequent cellular repair mechanism, these double-strand breaks can be utilized to create different gene editing varieties [78].

**Fig. 1:**
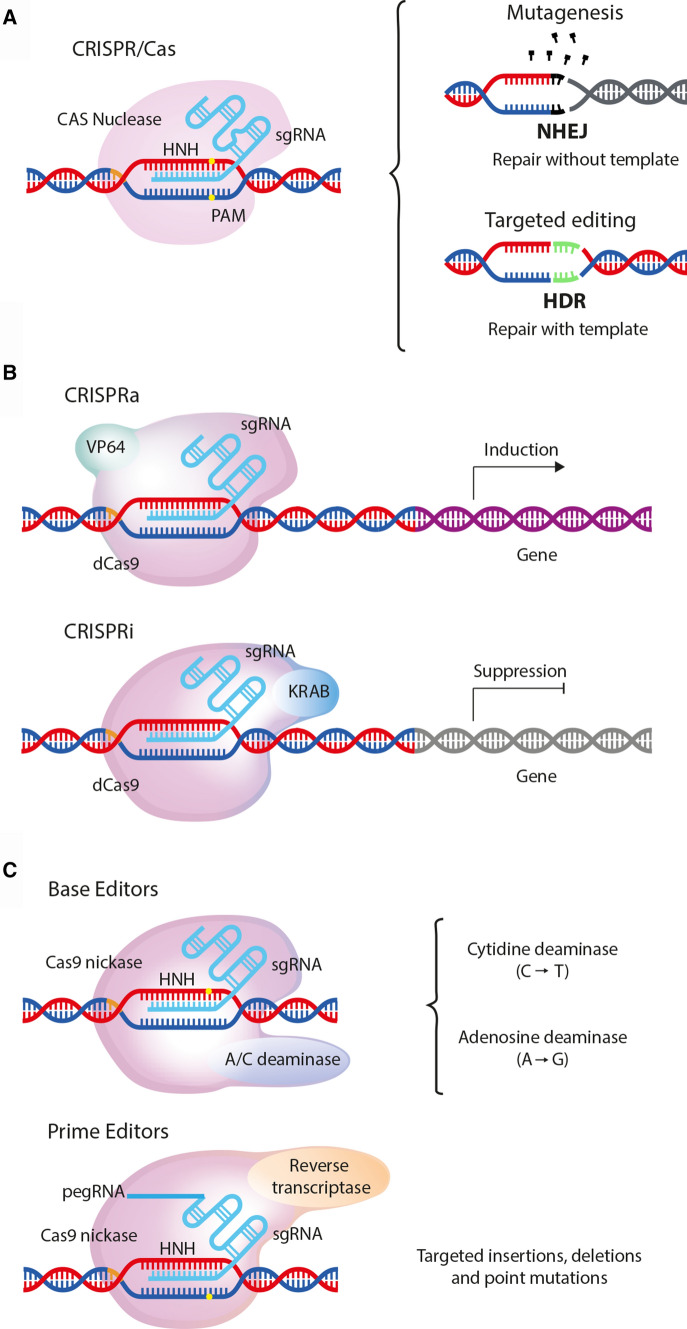
Principle CRISPR/Cas systems. *NHEJ* non-homologous end joining, *HDR* homology-directed repair, *PAM* protospacer-adjacent motif, *Cas9* CRISPR-associated protein carrying two nuclease domains, associated genes, *dCas9* catalytically dead Cas9, *Cas9 nickase* a Cas9 carrying only one nuclease domain to induce single-strand breaks, *sgRNA* single-guide RNA, *pegRNA* prime editing guide RNA, *VP64* gene inducer protein domain, KRAB gene suppressor protein domain

One of the most widely used CRISPR-associated enzymes is Cas9, which originates from *Streptococcus pyogenes* (SpCas9). SpCas9 forms a protein–RNA complex, resulting in cleavage of double-stranded DNA at target sites. SpCas9 is guided to this site by a single guide RNA (sgRNA), which is formed by the mature CRISPR-RNA (crRNA) and a trans-activating tracrRNA, and requires the simple protospacer-adjacent motif (PAM) NGG, to which the dsDNA cleavage occurs ~ 3 base pairs 5′ of the PAM (reviewed in [7]). DNA cleavage is followed by reparation of the DNA strand breaks by the cell, either through non-homologous end-joining (NHEJ) or homology-directed repair (HDR) mechanisms (Fig. 1a).

### CRISPR/Cas applications

For gene editing of cultured cells by the CRISPR/Cas system, most commonly plasmid-based or lentivirally guided expression methods are used [118, 125]. These systems are continuously and rapidly improved in specificity, efficacy, and applicability. For example, the Cas9 nickase mutants (D10A or H840A) can produce single-strand breaks [65, 94, 118]; other additions are high-fidelity Cas9 enzymes [72], an expanded PAM Cas9 variant called xCas9 [59] and Cas9 variants with improved proof-reading enhancing homology-directed repair (HDR) [68]. Systems for temporal and inducible control like photo-activatable CRISPR/Cas9 systems [106, 114] or doxycycline-inducible genome editing [41] have been developed and user-friendly bioinformatic tools have become available to design specific guide RNAs [34].

Addgene, a nonprofit plasmid repository (www.addgene.com), is of great help in the orientation phase of researchers as it provides an overview for what other purposes than knockout, knockin, and base editing CRISPR/Cas can be used. Especially the invention of the catalytically inactive dead Cas9 (dCas9), which lacks endonuclease activity and therefore does not cleave, but still can be guided to a target DNA sequence [115], opened a new field of alternative applications. One of such applications is CRISPR-mediated control of gene expression by, e.g., CRISPR interference (CRISPRi). Here, expression of targeted genes is efficiently repressed by either blocking or repressing transcription with a dCas9–KRAB fusion protein [48, 115]. In CRISPR activation (CRISPRa), dCas9 is fused to transcriptional activators (like VP64) to activate gene expression of the targeted gene [40] (Fig. 1b).

dCas9 fusions with epigenetic modifiers can be used to alter locus-specific epigenetic modifications like DNA methylation, histone methylations, and acetylations [40]. Dynamic live cell chromatin imaging profits from fluorescently labeled dCas9 to visualize genomic loci in a single-, dual-, or multicolor way [29, 91, 92, 116]. Interestingly, recent similar developments were initiated focusing on Cas13a/C2C2, Cas13b or CasRx/Cas13d, or their catalytically inactive variants. Importantly, Cas13 enzymes bind RNA and not DNA, which opens up new ways to study RNA biology [5, 32, 75, 119].

Specifically, for gene editing purposes, fusion proteins of dCas9 with other enzymes have been developed: for base editing without double-strand breaks, dCas9 was fused to cytidine deaminase, which mediates the direct conversion of C → T (or G → A) [12, 74]. This technique is more efficient (between 10 and 30%) than the knock-in approach and generates fewer unspecific indels [171]. Fusion proteins of the Cas9 nickase domain with reverse transcriptase allow a specific insertion of a sequence contained in the provided RNA into the genome and is called Prime editor approach [11, 74] (Fig. 1c).

### Clonal expansion and mixed culture

The CRISPR toolbox is currently growing rapidly [154] increasing its versatility, usability, and specificity [12, 49]. CRISPR techniques in general can be established in most labs and are fairly cheap, as many of the required plasmids are provided to the scientific community at no cost. An important hurdle, yet, is the introduction of the Cas and the gRNAs into the target cell. For this, direct injection, plasmid-based transfection or virus-based transduction is required. Cell lines usually have to be subjected to antibiotic-based selection to enrich the transfected cells, a step often followed by clonal expansion [169]. This workflow, however, is not applicable to primary cells. Such cells have a limited growth capacity before entering a senescent state and de-differentiate in culture. Moreover, when primary cells become too sparse, they often stop growing, so that the culture does not recover from the antibiotic-based selection. Thus, although clonal expansion might be a possibility for some cell types, it comes with the price of substantial phenotype alterations during the process. Obviously, for non-dividing cells, selection and expansion are not an option at all, and thus, for primary or non-dividing cells, systems with high transfection efficiency are needed. Viral delivery systems such as vectors based on adeno-associated virus (AAV), adenovirus, or lentivirus are able to transduce non-dividing cells [84]. For cardiomyocytes, AAV vector delivery enables HDR in murine adult heart tissues and human cardiomyocytes differentiated from induced pluripotent stem cells (hiPSCs) independently of the cell cycle stage [73]. In contrast to AAV, for adenovirus (AdV) vectors, genomic integration is rather an exception. In cultured neonate cardiomyocytes, however, some limited integration was observed for cells which had entered S-phase [73]. Viral vector systems vary in their packaging capacity, the genetic material (DNA/RNA), and the vector genome form. Adenoviral vectors (HCAdV) are rather effective in transduction and have a large packaging capacity. They can carry within a single vector a whole CRISPR/Cas9 system with gRNAs. The system has been successfully used for targeting the human papillomavirus (HPV) 18 oncogene E6, the dystrophin gene causing Duchenne muscular dystrophy (DMD) and the HIV co-receptor C–C chemokine receptor type 5 (CCR5) [42]. Very popular vectors are adeno-associated viruses (AAVs). They have already been approved for a number of human clinical trials, showed only mild toxicity at high doses in animal experiments, and are less immunogenic than other viruses. Most importantly, AAV displays a safe integration pattern and long-term persistence in non-dividing cells mediating stable gene expression [167].

Regarding the turnout of the gene editing events, the impact on the cell population will be diverse. Non-edited cells, successful edits, failed edits with chromosomal aberrations, and off-target edits will all be present in the small culture dish. As a result, in the mixed culture, the functional consequences of gene editing on the cellular phenotype will not be as pronounced as with clonal expansion. It should, however, be mentioned that clonal expansion is only superior to the mixed culture approach if several clones are being characterized and if the clones are carefully studied for the above-mentioned limitations. Whereas demonstration of the anticipated gene editing event is usually a relatively easy task with PCR, the demonstration of the absence of off-target editing events is laborious and expensive. Clonal expansion is also more prone to artefacts than a mixed culture approach. A recent study by the Odom lab, comparing different loss-of-function methods (siRNA, LNA, CRISPRi) showed that the introduction of the dCas9-KRAB protein alone had already strong effects on the transcriptomic landscape on a clonal level, whereas the non-clonal cell line showed almost no differentially de-regulated genes compared to the parental cell line [144].

The decision on the most appropriate technique for the individual purpose should therefore consider the type of cell, the transfection efficiency, and the quality of the gene editing approach. If the particular cell model under investigation permits the expansion of single cells and if efficacy of the sgRNAs is an issue, then clonal expansion may be considered. If the cell system does not permit clonal expansion or cannot refrain from natural heterogeneity of, e.g., a primary cell population, a polyclonal design may be chosen [128].

### CRISPR/Cas screening

**Fig. 2:**
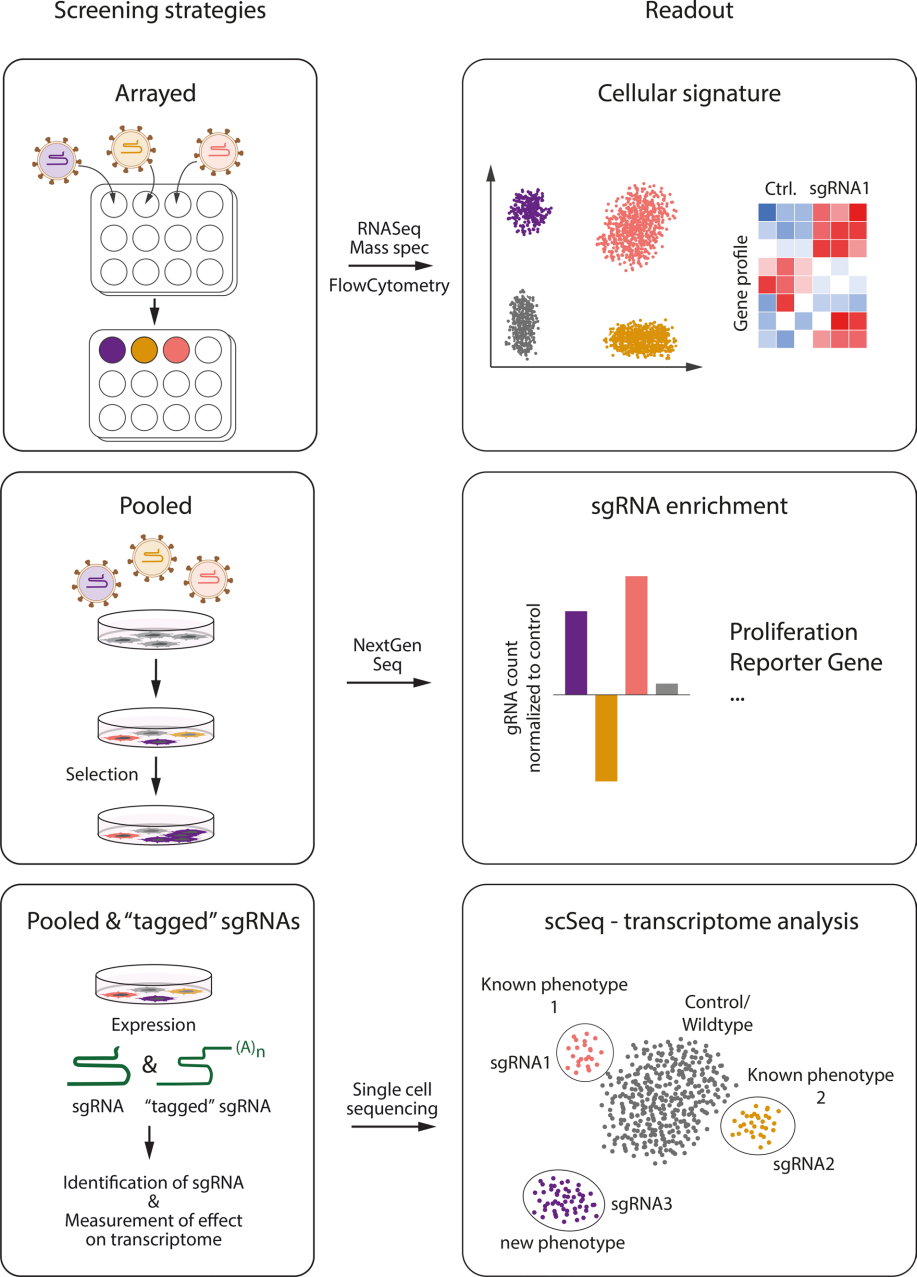
CRISPR/Cas screening possibilities. Three strategies have been developed for CRISPR/Cas screens: in array screens, single wells/dishes of cells are infected with one individual sgRNA each, the readout is typically on cellular signature signals, such as comparing bulk transcriptomes or performing surface protein expression profiles. Pooled screens involve transducing several to many sgRNAs and applying a positive or negative selection on transduced cells. Target genes that generate the desired phenotype are uncovered by deep sequencing and subsequent ranking of measured sgRNAs. Third, cells expressing any kind of CRISPR/Cas machinery are transduced with a pooled sgRNA library. Cells will express a functional sgRNA copy together with a second copy of the sgRNA that allows identification by sequencing [barcoding, poly(A)-tailing, e.g., used in, e.g., Perturb-Seq, CROP-Seq etc.]. After droplet sequencing, sgRNA-mediated perturbation can be analyzed in single cells. Multiple sgRNAs against a target gene are used to validate the phenotype

Screening technologies are used to uncover dependencies and relations targeting a number of interactors followed by the acquisition of a specific readout. In unbiased genetic screens, expression of a large array of genes is altered randomly, and the subsequent consequences on expression of other genes or functional parameters like proliferation or differentiation are determined. The CRISPR/Cas system has revolutionized the technology of genetic screening as unbiased forward and reverse genetic screens with large sgRNA libraries targeting thousands of genes in parallel are now possible. This technique currently represents the most powerful approach to identify genotype-to-phenotype relationships. To call these interactions, current screens take advantage of pioneering RNA interference work performed in drosophila [100]. Generally, there are two types of genetic screens—positive and negative—in which phenotypes and genotypes are either enriched or depleted, respectively [39]. These screens can be performed in arrayed and pooled conditions (Fig. 2). Arrayed screens have the advantage of physically separating genotypes, enabling subcellular and morphologic read-outs, but have the disadvantage of becoming quickly unfeasible when automated plate handling and phenotype recording is not available [8]. In contrast, pooled screens have the advantage that hundreds of thousands of genotypes can be tested simultaneously in adherent, suspension, or three-dimensional culture as well as living animals. However, read-outs of pooled screens are currently limited to cell fitness effects or depend on phenotypic reporters coupled to cell enrichment steps [98, 124].

The groups of Feng Zhang, David Sabatini, and Eric Lander were the first to report the use of CRISPR technology for unbiased genetic screening. In these pioneering studies, the identification of essential human genes and 6-Thioguanin and vemurafenib resistance mechanisms served as a proof of concept and represented the beginning of the CRISPR screening revolution [131, 159]. Since these early reports, technical parameters defining library composition (gRNA design rules and number of gRNAs per target) and optimal screening conditions [multiplicity of infection (MOI) and library representation (coverage)] have been identified [108, 127]. Currently accepted conditions include the use of 4–6 highly active gRNAs per target gene, delivered to cells with an MOI of 0.2–0.5 and represented in the experiment with 500–1,000-fold coverage. Standardizing these conditions enables the comparison of different gRNA libraries and their in-screen performance in different cellular model systems. This has vastly contributed to the increase in screening reproducibility [16, 67, 108, 153]. The majority of reported CRISPR screens were designed to identify novel gene to phenotype relationships, while only few studies investigated functional aspects of non-coding sequences [23, 134, 160]. Strategies to efficiently interrogate and functionally annotate non-coding regions apply either DNA tiling or excision approaches. For both approaches, genomic regions are targeted by all possible gRNAs within a given region or the DNA connecting two juxtaposed gRNA-target sites is excised and lost, respectively [23, 76, 126]. Tiling approaches were initially used to identify functional domains in proteins, but have since been used successfully to find essential nucleotide sequences in predicted promoters, enhancers, and long non-coding RNAs [87, 126, 132]. Excision screens are currently limited to a few target sites, mostly because no technological solution exists to generate pooled gRNA libraries with predetermined gRNA combinations [130]. Noncoding sequences make up the vast majority of the human genome; hence, it will be important to solve this technical issue to enable broader unbiased investigations into this “dark” region of the genome.

For CRISPR screens, data recording is robustly and routinely performed by next-generation-sequencing (NGS), while data analysis lacks a broadly accepted approach. Several algorithms (e.g., MAGeCK, HitSelect, PinAPL.py, and ScreenBeam) with differing statistical assumptions have been developed, though it is currently unknown which pipeline performs most accurately as data on comparing hit validation are still missing [37, 82, 142]. Also, most of these tools have been repurposed from the analysis of RNAseq data and only few algorithms are designed for the needs of CRISPR screens. With the increasing number of CRISPR screens that aim at identifying gene–gene interactions, CRISPR-tailored data analysis algorithms are needed. The search recently culminated in the GEMINI algorithm to identify genetic interactions in multiplexed or combinatorial CRISPR screens [170]. As the CRISPR screening field is moving towards multiplexing, it will be crucial to establish, similar to single screens, experimental parameters and statistical models that enable high reproducibility and hit penetrance [38].

### CRISPR/Cas screening in combination with scRNA-Seq

The recent rise of single-cell RNA sequencing (scRNA-Seq) permitted for further evolution of CRISPR-based genetic screens [39, 112]. While pooled screens produce rather simple, low-dimensional read-outs such as “growth” or “no growth” and typically require validation by further experimentation, scRNA-Seq of CRISPR-manipulated cell populations employ the transcriptomic signatures of individual CRISPR-manipulated cells as high-dimensional, complex phenotypes (Fig. 2). The assay principle relies on the introduction of “barcode” sequences (or a polyadenylated copy of the sgRNA itself) together with individual sgRNA sequences, thus permitting the assignment of gene expression profiles to the manipulation of defined genetic loci in one single experiment. A treatment or selection is not necessarily needed to perform such screens (although the frequency of the sgRNA still can yield some insight on, e.g., lethality of a knockout/knockdown) (Fig. 2).

### Editing the genome of hiPSC for advancing disease modeling

The advent of human-induced pluripotent stem cell (hiPSCs) technology has provided a huge opportunity to establish cellular models of disease from individual patients, and to study the effects underlying genetic aberrations of inaccessible cell types [93, 141, 147]. Particularly, the investigation of molecular mechanisms and cellular phenotypes resulting from a specific mutation (rather than the individual’s genetic background) is possible in iPSC models of disease through the correction of mutated genes in diseased iPSCs (isogenic controls), or by the introduction of causative mutations in healthy iPSCs [17, 140]. It should be emphasized that, contrary to monogenic diseases, the dissection of polygenic disorders is more complicated as a large number of genetic variants are acting in a complex network, and a single variant is not sufficient to trigger the disease [97, 99]. A specific challenge is posed for diseases involving X-linked genes. When using hiPSCs to model X-linked developmental disorders or inherited conditions that undergo sex-specific modulation of penetrance (e.g., autism spectrum disorders), it is crucial to consider the course and status of X chromosome inactivation (XCI). XCI is a unique dosage compensation mechanism that occurs during early embryogenesis and enables equivalent expression of X-linked genes between male and female mammals [56]. Reprogramming of female cells can give rise to two different hiPSCs: XCI has been maintained as in the original somatic cell, or X-chromosomal reactivation (XCR) occurs followed by random mosaic or skewed XCI in the differentiation state, according to the pluripotent state (primed versus naïve) [31, 80, 123]. A third rare outcome is an abnormal partial XCR, in which differentiation does not yield a fully XCI [149]. The failure to precisely characterize XCI status can have significant consequences for the validity of hiPSC-based disease models and their implementation after genome editing approaches.

Among genome editing technologies, CRISPR represents the most electable approach in many cell and tissue types [18, 65, 66, 74, 155]. As mentioned earlier, targeted dsDNA cleavages are repaired through two different pathways: NHEJ and HDR. NHEJ results in insertion and deletion (indel) mutations and can be employed to insert a premature stop codon, resulting in knockout of the gene. This strategy has been recently used to study the deficiency of the KCNA5 gene, which leads to a lower beating rate and prolonged field potential durations of atrial cardiomyocytes [96]. The same approach could be used to restore the normal activity of genes, for instance deleting expanded CGG repeats of the FMR1 gene to rescue Fragile X syndrome [111]. The combination of CRISPR and the PiggyBac transposon systems enables the insertion of large modifications needed in cases of large deletions such as Duchenne muscular dystrophy and Huntington’s disease [86, 165].

HDR-mediated genome editing repairs DNA in a precise manner referring to the sister chromatid as template and can be used to introduce or correct specific disease-associated variants for disease modeling. Correction or introduction of disease-associated gene variants (1–20 nt) has recently been accomplished with the use of short single-stranded oligonucleotide donors (ssODNs) with homology arms of 30–200 nt [121, 168]. An advantage of ssODNs, besides their fast design and generation, is that they are less likely to integrate randomly into the genome in comparison to plasmids or linear dsDNA donors [83]. The ssODN strategy results in scarless editing, but the lack of selectable markers encoded within the donor increases the difficulty for identification of positive clones. Alternative to ssODNs, single-stranded rAAV DNA templates harbor the beneficial feature of providing long homology arms and resistance genes for increased recombination efficiencies with low rate of genomic integration. Moreover, CRISPRa/i approaches are useful in treating human diseases due to haplo-insufficiency or protein accumulation such as Parkinson and Alzheimer’s disease [57].

Overall, genome manipulation of hiPSCs has become highly efficient using CRISPR, especially for NHEJ-based pathways (80–90% with NHEJ vs < 10% with HDR). Nonetheless, Cas9 can bind off-target sites with mismatches, resulting in variable off-target activities [151]; Cas9 construct is either permanently integrated [24, 50] or removable with a subsequent reagent delivery and/or clonal selection step [164] to achieve scarless editing. Alternatively, non-integrating methods include Cas9 ribonucleoprotein (RNP) complexes, which are immediately active and rapidly degraded over a period of around 12 h [69], reducing the potential for off-target mutagenesis and re-targeting after successful HDR.

Repairing causative lesions in patient-derived iPSCs which are subsequently differentiated towards specific cell types might be used for cellular therapy treatment. Some strategies are promising, *e.g.,* injection of autologous iPSC-derived dopaminergic neurons in the treatment of a chemically induced primate model of Parkinson’s disease [55]. Nevertheless, various technical hurdles and biological questions need to be addressed before the introduction of therapeutic use of CRISPR/Cas in humans.

## Genome editing in animals

### Genome editing and disease modelling in zebrafish

**Fig. 3:**
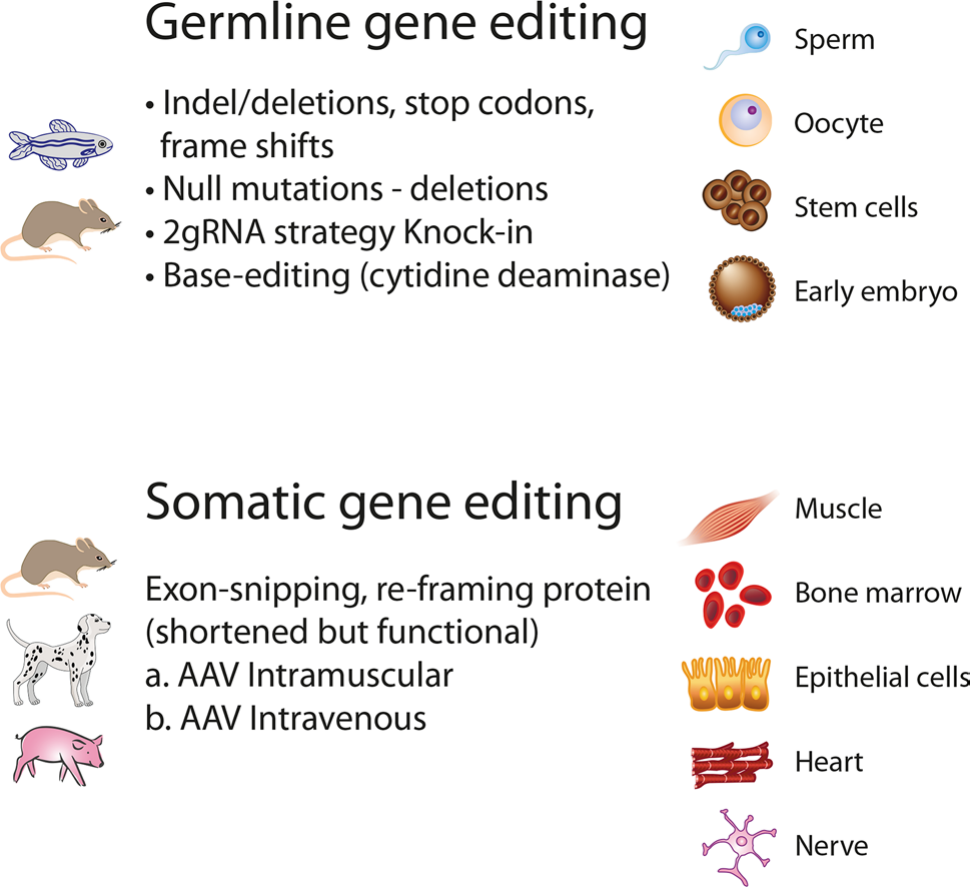
Domains of gene editing in animals. Whereas Cas9-mediated germline gene editing now becomes the standard technology for the generation of transgenic fish and rodents, larger mammals, like transgenic pigs are still generated by “conventional” technology. Pigs and dogs are important laboratory animals in the translational avenue to established somatic gene editing for clinical use

TALEN and CRISPR/Cas9 are meanwhile the standard tools for reverse genetics in zebrafish (Fig. 3). Originally, Cas9 and a single sgRNA were used to target a specific region within the gene of interest forming insertion/deletions (indels), premature stop codons, or frameshifts, which inactivate the generation of a functional gene product [26, 35, 60, 95]. Depending on the position of the premature stop, nonsense-mediated decay of transcribed RNA and production of small RNA fragments may trigger the expression of genes that can compensate for the mutation and mask the phenotype [44]. Such compensation mechanisms, which are also operational in mice, account for the discrepancy between morpholino knockdown and mutant phenotypes. The current consensus for generating a mutant therefore is to suppress the RNA transcription of the target gene completely, thereby avoiding the formation of RNA decay products and compensation. This is achieved by either deleting the full gene locus, or by removing the complete transcriptional or translational start regions using a combination of two sgRNAs. In general, control experiments suitable to uncover potential compensation do not target the gene but rather its expression, like CRISPRi or shRNA.

CRISPR/Cas9 is also used for knock-in approaches in zebrafish [14, 19, 70]. The knock-in can be utilized to generate specific—patient similar—mutations, or repair existing mutations [148]. The current limitations of this technique are the low efficiency (between 0.5 and 1%) and the additionally generated off-target mutations. Base-editing, for example by a fusion of dCas9 with cytidine deaminase, has a higher efficiency (10–30%) and greater specificity [171].

### Genome editing and disease modelling in mice

Similar to zebrafish, also in mice, the *CRISPR/*C*as9* system replaced classical genetic engineering techniques to generate different genome-engineered transgenic mouse lines [52, 78, 89]. Cas9 is the most widely used nuclease due to the simple protospacer-adjacent motiv (PAM) 5′-NGG-3′ and the generation of double-strand breaks (DSB) compared to Cas12a (Cpf1), which has a PAM (5′-TTN-3′) that is T-rich, less frequent in the genome and creates staggered ends [28, 107]. Among the mutations generated are classical and conditional gene knock-outs (KOs), knock-ins (KIs) at specific genomic loci as well as point mutations or epitope insertions in desired gene regions. The efficiency of gene modification at specific loci using the NHEJ mechanism reaches almost 90% and the efficiency of KI using the HDR mechanism is up to 50%. To increase HDR frequency, different strategies to inhibit NHEJ and to enhance DNA repair mechanisms are implemented (for review, see [35]). Nevertheless, these rapidly evolving techniques still contain serious limitations exemplified in several KI experiments by the creation of conditional KO mouse alleles. All gene-targeting protocols are performed by direct injection of CRISPR/Cas9 components together with donor DNA template into fertilized oocytes. For KI experiments, comparatively long-donor DNA fragments (~ 600–1650 nt) are utilized employing either single-stranded (ssDNA) or double-stranded (dsDNA) templates. Efficiency of homologous donor DNA template integration into the locus is variable and correlates with template size and the locus. In general, longer DNA templates integrate less efficient than shorter ones.

Attention should be paid to the fact that most genome edited mice obtained from CRISPR/Cas9-modified zygotes (F0 generation) exhibited mosaic genotypes. The mosaic genotype can harbor subpopulations of germ cells derived from different DNA-editing events, and contain diverse copy numbers of DNA template integrations into the targeted loci [136]. The latter suggests that PCR amplification of short flanking genomic regions together with parts of the inserted artificial sequences, including the LoxP sites in template DNA, is the most efficient and reliable approach for the identification of F0 mice with a correctly targeted event. When the selected F0 founders were crossed with wild-type mice to obtain F1 offspring, animals harboring multiple head-to-tail integrations (MHTI) of the donor DNA template at the targeted locus can be detected frequently. These DNA template multiplications occur irrespectively of the size, nucleotide composition, or the utilization of dsDNA or ssDNA [136, 138].

Importantly, a commonly used PCR analysis method of heterozygous animals, employing locus-specific oligonucleotides located outside of the targeted homology region, would under this condition, in most cases, mistakenly indicate a single-copy integration event. Southern blot analysis is an efficient accompanying method to reliably identify the desired single-copy targeted events in F1 offspring. It is recommended to include two different specific restriction endonuclease sites flanking the *LoxP* regions. This will allow the detection of correctly targeted events by restriction fragment length polymorphisms (RFLP), using these restriction enzyme sites, and also detects the MHTI. In addition, a PCR approach with reverse orientation of DNA template-specific primers, qPCR, or the ddPCR (droplet digital PCR) can be alternative methods for detection of MHTI of the DNA template [136]. To exclude any genome alterations, sequencing of the entire locus is required.

### Genome editing and disease modelling in large animals

Large animals are important model organisms to bridge between basic science studies and clinical applications. In the cardiovascular system, this has been exemplified for mutations of the dystrophin gene leading to Duchenne’s muscle dystrophy (DMD). A number of mouse models have demonstrated efficacy of an AAV-based CRISPR/Cas9 approach to edit the dystrophin gene. In the *mdx* mouse, excision of the mutated exon 23 suffices to enable expression of a shortened, but stable dystrophin gene [43, 88, 105, 146]. Functional assessment, though limited due to a mild phenotype of the mouse model, suggested improvement of the skeletal and heart muscles after local or systemic vector application.

Extending this evidence, Amoasii from the Olson lab [10] applied single-guide RNAs and SpCas9 AAV into DMD dogs (lacking exon 50), either intramuscularly (i.m.) or intravenously (i.v.), and found robust DMD expression at the injected sites (i.m.) and also in the heart (i.v. approach). Functional consequences of this approach though were not reported yet.

In a complementary transgenic pig model lacking exon 52 of the dystrophin allele, an intein-split version of SpCas9 [150] was used together with a vector containing a pair of gRNAs capable of excising exon 51 also using AAV technology [103]. The intein-split approach bypasses the packing limit of AAVs: two virus particles are generated which both carry a gRNA and the C-terminal and N-terminal, respectively, part of Cas9. Only after a double infection of a cell, a complete Cas9 is produced [150]. After i.m. injection, the intein-split-Cas9-gRNA approach was efficient in editing up to 78% of the muscle nuclei analyzed. Upon i.v. injection, 7% of the cardiac genomes were edited, resulting in a reduction of sudden cardiac death of the animals [103].

### Delivery of CRISPR/Cas9 in somatic tissue

Besides off-target activity which will be discussed in the next section, the aspect of a safe and efficient delivery of the gene editing tools is crucial in a therapeutic context. Although a number of different approaches have been developed to facilitate direct Cas9 ribonucleoprotein complex delivery by utilizing nanoparticles, extracellular vesicles, or cell penetrating peptides (for review, see [22, 166]), many therapeutic approaches are relying on the delivery of genomic encoded tools via viral systems (Fig. 3). In this context, the AAV system is advantageous due to its capability to transduce both proliferating as well as post-mitotic cells, its diverse tissue tropism, its robust and prolonged expression levels, and its relatively low immunogenicity [13]. Nevertheless, the maximum packaging capacity of about 4.7 kb resembles a limitation for single vector approaches, especially if the relatively large *Streptococcus pyogenes* variant of Cas9 is used. To overcome this limitation, either the co-administration of a Cas9 virus plus a separate gRNA expressing virus or the utilization of a split-Cas9 system can be helpful [13].

**Fig. 4:**
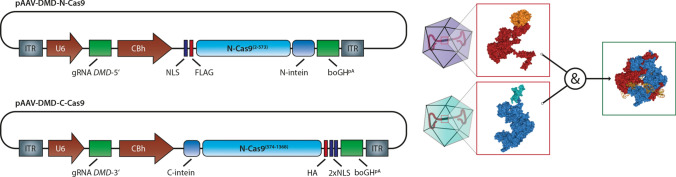
Split-Intein-AAV-System: due to the packaging limit of adeno-associated virus (AAV), the most-frequently used Cas9 genes together with two guide RNA cannot be transducted by a single AAV. In the Split-Intein-System, the cell is infected with two different viruses, both containing one part of Cas9 and one part of the Intein gene. Expression of both constructs yields a complete, enzymatically active Cas9

The previously described therapeutic studies for DMD exemplify these different strategies: the group of Eric Olson applied a single cut approach to restore the reading frame of exon 52 deficient dystrophin by insertions or deletions (Indels) in the 5 prime region of exon 51, and partially also by enhanced skipping of exon 51. The group of Christian Kupatt follows a different approach utilizing the split-intein system (Fig. 4). Here, two distinct AAV constructs, each harboring one individual gRNA under the control of an U6 promoter and one half of the intein-fused SpCas9 nuclease under the control of a pol-II promoter, are co-injected. Upon co-expression in the same cell, the N- and C-terminal halves reconstitute and perform guided nuclease activity analogous to wild-type Cas9 [103]. This setup allows the delivery of SpCas9 together with two independent gRNAs and is only active in co-transduced cells. Instead of one gRNA located in exon 51, two gRNA in the intronic regions flanking exon 51 have been selected to precisely excise the exon and thereby restoring the reading frame. In general, these exon snipping approaches allow a more flexible design of the utilized gRNAs and thus enable a more stringent selection regarding predicted off-target activity. Furthermore, compared to full length Cas9 approaches, this system has the potential to operate under the combined control of two distinct, tissue and/or cell type-specific pol-II promoters, which further enhances not only the specificity but also the safety of the gene therapy approach. Apart from this, in general, every aspect of a gene therapeutic approach has to be optimized to achieve the highest level of safety. This includes an efficient and safe delivery of the tools, a specific expression of a Cas9—ideally restricted to the tissue/cells of interest and preferably a temporal restriction of the gene-editing event.

### Analysis of CRISPR/Cas9 off-targets

Beside the thorough characterization of the targets, off-target events deserve closer attention. Programmable nucleases such as CRISPR/Cas9 are efficient to generate on-target genetic modifications; however, rigorous design of sgRNAs, Cas9, and delivery modes are necessary to minimize potential off-target genomic alterations and to ensure the integrity of the genome of manipulated cells (for review, see [62]). To facilitate the use of crRNA, representing 20 bp complementary sequence to the target genomic region and tracrRNA (trans-activating crRNA) representing a scaffold to bind Cas9 both have been combined into a sgRNA [66]. It has been reported that even 3–5 mismatches at the distal (5′) end of the protospacer sequence can be tolerated leading to DSB [46]. Thus, if sgRNAs are not properly designed, off-target effects might be induced.

To minimize off-target activity and increase specificity, truncated guide sequence at the distal 5′ end [45] was shown to be beneficial so is chemical modification at the 3′ end of gRNA [102]. In addition, a number of web tools have been designed for proper design of gRNAs taking also PAM sequences into account and to identify potential off-targets genome wide, e.g., CRISPRdirect [104], Cas9 design [90], and ChopChop [39, 101]

Based on the three-dimensional structure of Cas9, mutants (D1135E) have been generated which increase on-target specificity and recognize [71] different PAM sequences of different lengths, thereby increasing specificity. Furthermore, Cas9 nickase mutants have been developed in which the RuvC or HNH nuclease domain is inactivated resulting in juxtaposed single-stranded nicks [79], or a pair of catalytically inactive dCas9 nucleases each fused to FokI nuclease domain [26, 113]. Each of these approaches reduce off-target mutagenesis; however, they also have their limitations with respect to cleaving efficiency, e.g., double nicking requires two guides and truncated guides can reduce on-target efficiency, as well. Using structure-guided protein engineering in combination with unbiased whole-genome off-target analysis, enhanced efficiency variants of *Streptococcus pyogenes* Cas9 (eSpCas9) have been generated which exhibited reduced off-target and robust on-target effects [137].

Reduction of off-target mutagenesis can also be achieved via temporal expression of Cas9 taking advantage of a tetracycline responsive promoter [137], using split-Cas9 intein system [137], and by inactivating Cas9 through self-cleavage providing in addition Cas9-specific gRNA [137].

Different methods are available to search and inspect potential off-target sites. There are three main strategies available: (1) to determine sequences of potential off-target sites; (2) to determine potential DSBs genome wide; (3) to determine genome integrity.

The first straightforward strategy is to determine predicted off-target sites using software and assay the PCR products for mismatches by single-strand annealing and endonuclease treatment (e.g., T7EI, Surveyor nuclease assay). These techniques are most commonly used as they are easy to implement and cost effective. Alternatively, deep sequencing of exome genome wide (all protein coding regions) is performed to identify potential mutations in protein coding genes [148]. A more in-depth analysis is achieved by whole-genome sequencing (WGS) used in cell lines [139, 145, 156] and mice [64]. These techniques are powerful and allow to identify small indels but not large genomic rearrangements. Larger genomic rearrangements and CNVs need to be examined using alternative methods, e.g., fluorescence-in-situ-hybridisation [20].

Second, identifying potential DSBs by chromatin immunoprecipitation and pull-down of DNA fragments (ChIP-seq) have been employed in different flavors, e.g., using dCas9 to determine Cas9-binding sites [162]. Alternatively, strategies have been developed which label DSB by either streptavidin-biotinylated linkers [33] or by incorporating short phosphorylated double-stranded oligodeoxynucleotides (GUIDE-seq [152]). An elegant extension of these strategies is the DISCOVER-seq (discovery of in situ Cas off-targets and verification by sequencing) method that leverages on the recruitment of DNA repair factors in particular MRE11, which binds closely around the Cas9 cleavage site, to uncover Cas9 activity [161].

Additional technologies are useful to confirm the results obtained by CRISPR/Cas9: Despite having their own potential off-target effects, RNA interference with different siRNAs/shRNAs, the use of different (morpholino) antisense oligonucleotides, and LNA GapmeRs, as well as different pharmacological inhibitors should be taken into consideration to validate on-target effects seen by CRISPR/Cas9-mediated knockout or inhibition experiments and to further rule out the possibility of off-target effects. In the case of CRISPR/Cas9-mediated activation systems, plasmid- or viral-overexpression systems can be used for clarification. Moreover, as mentioned before in the section regarding clonal expansion, but not only concerning mixed populations, several clones of the control and the mutants should be characterized carefully to rule out off-target effects and to strictly define the limitations of the systems. Another way is to test directly the expression of genes which were predicted in the gRNA web design tools. For genome editing in mice, back crossing the transgenic mice generated by CRISPR/Cas9 could help to reduce the chance of off-target effects.

Thus, as with other genome and nucleic acid interfering techniques, we should be cautious with results obtained by CRISPR/Cas9. A broad set of tools is already available to reduce and detect off-targets and maintain robust on-target mutation. Selection of the strategy depends on the experimental setup, e.g., cell lines, animal models, preclinical gene therapy models, and whether an ex vivo or in vivo gene therapy approach is taken. In case of genome editing in model systems, potential off-targets can be reduced simply by back crossing wild-type animals to the F2 generation. In addition, at least two independently established mutants should be phenotypically characterized. For an in vivo gene therapy approach, rigorous experimental design and pretesting of gRNA, ideally in patient-derived cells, are required as well as applying high-end design, optimized endonucleases, optimized delivery strategies, and validation tools to minimize potential off-targets. Irrespective of all precaution measures, there is still an unforeseen risk of generation of by-stander mutations in the genome.

## Considerations for clinical gene editing

**Fig. 5:**
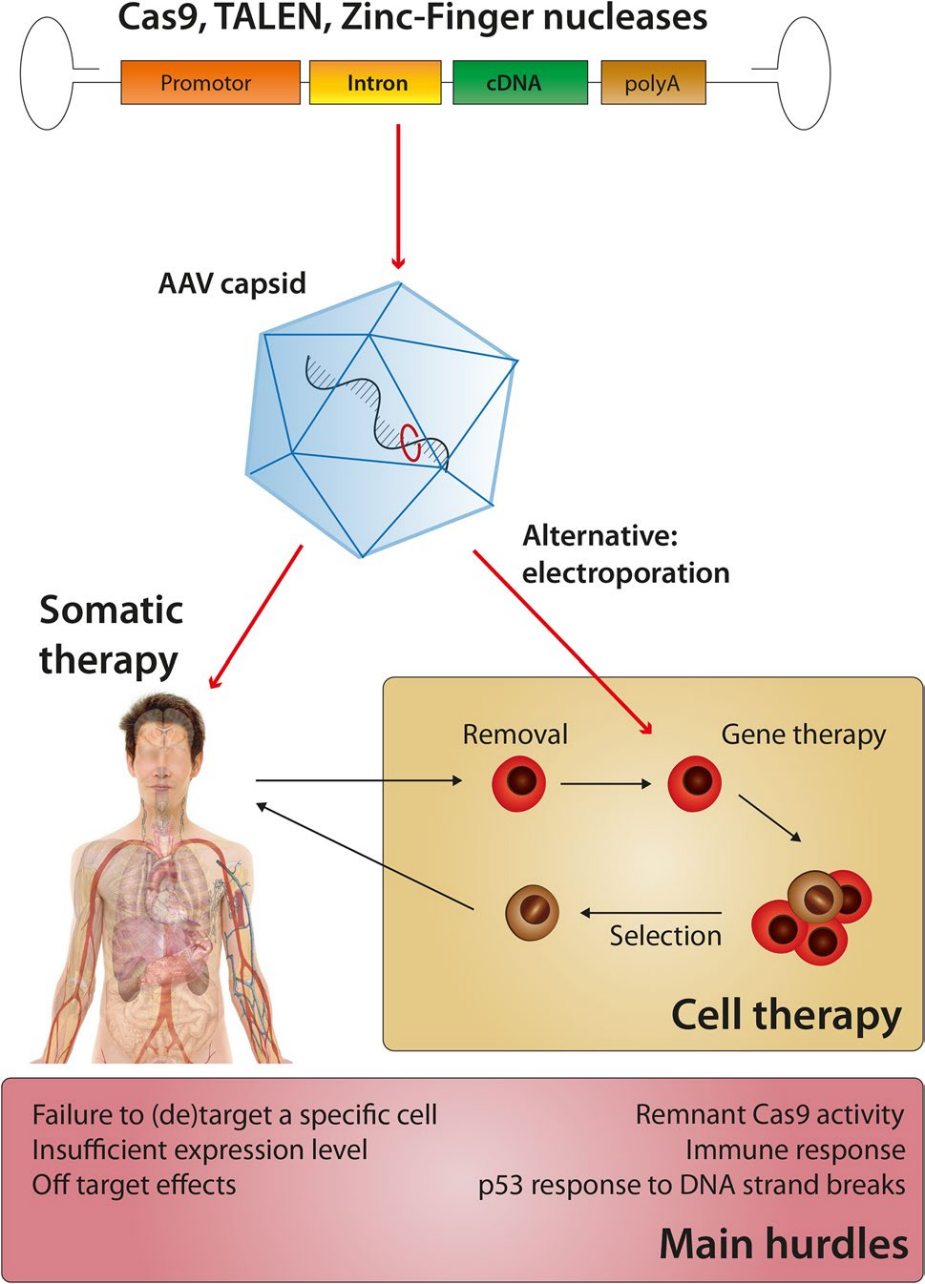
Concepts of human genome editing

Clinical translation and thus disease treatment are the ultimate aim of many gene therapy approaches. Several clinical studies with zinc-finger nucleases or later on TALENs have been initiated since 2009. They focused on a variety of conditions such as cancer, HIV, and hematological diseases with the outcomes still to be reported in most cases [84, 109]. In the last years, first clinical trials with CRISPR/Cas9 approaches have started to recruit patients (Fig. 5). Importantly, recent preliminary results from a clinical phase I trial showed that gene editing using CRISPR/Cas9 might be safe and feasible to apply [143]. T-cells from three patients with different types of advanced cancers were gene edited by CRISPR/Cas9 ex vivo using electroporation, resulting in ablation of three proteins that could inhibit the T-cells’ ability to target tumor cells. In a second step, a cancer-specific T-cell receptor transgene was expressed in these cells by lentiviral gene transfer to recognize a particular epitope on tumor cells. After administration into the respective donors, gene-edited T-cells were engrafted and persisted for at least 9 months without significant side-effects. As cancer continued to progress in all three patients, the question of efficiency of the gene-edited T-cells against advanced cancer remains open. In this particular trial, gene disruption efficiency was 15–45%, based on the techniques available at the time the study was approved (2016), whereas newer techniques allow now > 90% of gene disruption [122, 129]. Apart from the preliminary CRISPR/Cas9 clinical data, further gene editing studies in patients have been initiated aiming at elimination of mutations that lead to the development of cancer or hereditary diseases such as sickle-cell anemia, beta-thalassemia, or Leber congenital amaurosis [84]. The selection of human diseases currently treated with gene editing approaches, however, also illustrates the current bottleneck of clinical gene therapy: delivery of the CRISPR/Cas9 system and the sgRNA in patients.

While efficiency is already a major hurdle in clinical trials of gene therapy in general and in particular for cardiac diseases [63, 85], specificity of gene transfer is of decisive importance for gene editing approaches considering potential off-target effects such as chromosomal translocations and rearrangements with at least a theoretical oncogenic potential. Therefore, current studies in patients predominantly focus on ex vivo gene editing (i.e., cell therapy), for example in hematopoietic stem cells for sickle-cell anemia or T cells for novel cancer therapies, where the gene-edited cells can be reinfused into the body. In addition, ex vivo delivery allows a transient transfer of Cas9 and sgRNAs by electroporation that results in almost an absence of residual Cas9 activity in the cell product [143]. This might be important not only to limit the duration of Cas9 activity for safety reasons, but also to avoid immunological clearance of the gene-edited cells due to development of a humoral response to Cas9.

It is not surprising that the first clinical study using viral vectors for delivery of Cas9 and sgRNAs in vivo aims at treating a particular form of Leber congenital amaurosis [4], in which the coding sequence of the defective gene is too large to be packaged in a viral vector for the conventional overexpression. The eye is an immune privileged and easily accessible organ which allows efficient subretinal injection of AAV vectors as previously shown in a successful clinical study: a classical AAV-based gene replacement strategy revealed improved vision in patients with congenital amaurosis due to deficiency of a protein required for photoreceptor function [15].

While rapid clinical translation of the CRISPR/Cas9 technology is impressive, several limitations need to be overcome before gene editing approaches can be applied for cardiovascular diseases. Target cells such as cardiomyocytes require an efficient in vivo delivery system consisting of a suitable vector (for example, AAV vectors), but also a suitable application system for transvascular gene transfer such as coronary venous retroinfusion for cardiac transduction [63, 117]. Considering that any transvascular vector application results in some systemic spill-over, unwanted side-effects need to be prevented by confining expression of gene editing elements to the target tissue, for example, by use of tissue-specific promoters or alternative detargeting approaches [47, 63]. Alternatively, ex vivo gene editing therapies of whole organs (i.e., heart or lung), for which short time artificial organ support is possible, can be a first step towards clinical application in cardiac or pulmonary diseases.

Even if gene transfer into less immune privileged organs such as the heart would be efficient with viral vectors and appropriate delivery systems, it is not clear whether Cas9—a bacterial protein—will trigger an adaptive cellular immune response. Furthermore, also preexisting humoral reactivity to Cas9, which was previously detected in serum of healthy individuals, could affect sustained expression of Cas9 in transduced cells [27, 135, 158]. Thus, it might be necessary for in vivo gene editing in less immune privileged organs to control Cas9 gene expression using inducible promoters or switch to vector systems that enable an only transient Cas9 expression.

Beside the immunological effects of the Cas9 enzyme, a further limitation towards broad clinical translation of the CRISPR/Cas9 system might be the p53-mediated response to double-strand breaks induced by CRISPR/Cas9, leading to a principally but not widely proven enrichment for P53-deficient and thus more cancer-prone cells [53, 61]. Moreover, target recognition is prone to error, so that gene editing may introduce changes at partly unpredictable off-target sites due to similarities to the target recognition sequence. Even more alarming is that repair of double-strand breaks induced by gene editing approaches leads to large deletions, insertions, or complex rearrangement events involving the target site [77]. These unintended edits are detected consistently [6, 110, 133, 163, 173] and highlight the need for accurate genotype characterization, as these potentially have devastating outcomes in clinical tests. The repertoire of molecular, genetic, and next-generation-sequencing techniques to analyze (CRISPR/Cas9-mediated) changes on the genome is large and sufficient for verification of editing success and prevention of unwanted events. The opinion article by Burgio and Teboul [21] provides assistance and advice for the application of CRISPR/Cas9. Accurate predictions and the understanding of the whole range of possible editing outcomes are critical for the continuous success of CRISPR/Cas9.

Numerous approaches to increase specificity of gene correction have been undertaken focusing on optimization of the gRNA or the nucleases used for gene editing itself [59]. These improvements include the development of small molecule tunable Cas9 enzymes [36]. Also new variants of Cas nucleases, such as xCas9 and SpCas9-NG, enable the targeting of alternative PAM sequences and thus expand the range of genomic loci that can be edited [10, 143]. Fusion of nucleobase deaminase enzymes to catalytically inactive Cas variants makes it possible to accomplish mutual conversion among four bases [51, 74, 81, 172]. These so-called “base editors” modify base pairs at specific sites, thereby expanding the potential applications of the CRISPR/Cas system to correct disease-associated single nucleotide polymorphisms. Cytosine and adenine DNA base editors (CBE, ABE) and their approximate editing windows have been described in [120]**.** Both could be useful tools also to install or correct pathogenic point mutations. CBEs mutate C•G-to-T•A by binding to genomic target regions and R-loop formation. They bind to a target DNA sequence and form a single-stranded R-loop, and the covered cytosines are deaminated to form uracil bases. Uracil glycosylases are inhibited by the uracil glycosylase inhibitor domain and the Cas9 domain regulates DNA repair leading to an adenine opposite the uracil. ABEs mutate A•T-to-G•C by deaminating target deoxyadenosines to deoxyinosines, regulating the direct DNA repair to install a cytosine opposite the inosine nucleoside [12]. A recent development called “prime editing” showed less off-target editing without introducing double-strand breaks or donor DNA by fusing a catalytically inactive Cas to an engineered reverse transcriptase [11]. Editing individual bases in RNA offers also great potential in medicine. Adenosine conversion to inosine, which is generated by the adenosine deaminases from the ADAR family, has been shown to be a useful tool using both antisense and Cas13-guided RNA-targeting methods [120]. If and when these improvements (see also Fig. 1) may enter clinical trials is not known.

In summary, development of therapeutic gene editing approaches requires a careful design of the genome editing strategy including the most appropriate Cas variant, an appropriate vector system, and mode of vector delivery.

## Clinical perspective of gene editing

Considering the fast pace of clinical translation of gene editing approaches and but also the many open safety questions, the question of the best way to clinical translation emerges. It is current consensus that clinical trials can be started now and that these should aim on advanced cancers or rare hereditary diseases, for which efficient gene delivery modes are well established. These early studies enable collection of safety data which is highly needed for further studies with improved gene editing tools.

It is important to stress that gene editing techniques—as all other advanced therapeutics—require continuous consideration of social, ethical, and regulatory issues. However, increasing commercial interests may cause conflicts of interest preventing unbiased dissemination of methods and results. Instead of being published in peer-reviewed journals, results of commercial research and trials executed by companies are rather disseminated in business statements and announcements on websites [25, 109]. However, full disclosure of methodologies and concise analysis of off-target effects will remain necessary to assess the impact of any novel gene editing approach and to establish trust for further clinical translations.

Although apparently technically feasible, there is broad consensus among stakeholders that gene editing needs to be restricted to somatic cells as there are significant ethical, scientific, and socio-economic concerns regarding germline genome modification [9, 30]. As for all gene therapy approaches, Germany and many other countries have issued a strict ban on application of techniques for the purpose of human germline modification.

Legislation of gene editing approaches in somatic cells follow those for gene therapy products. As ex vivo gene therapy requires reapplication of genetically modified cellular products into the patient, these products must also comply with both cell-based medicinal product and gene therapy product guidelines and regulation. Details regarding the regulations of gene therapy products are reviewed elsewhere [54]. In Germany, two studies investigating ex vivo somatic gene editing for treatment of beta-thalassemia (NCT03655678) [2] and sickle-cell anemia (NCT03745287) [3] have already been approved to be conducted.

To further improve safety of gene editing approaches, several organizations, including the World Health Organization, as well as recognized standards developing organizations such as the US National Institute of Standards and Technology Genome Editing Consortium, the US Pharmacopeia, and the International Organization for Standardization (ISO) formulate gene editing standards [9]. Such standards are urgently needed to address key concepts like off-target effects and their impact on tumor suppressors and oncogenes. In addition, national and supranational regulatory organizations such as the Food and Drug Administration (FDA) and the European Medicines Agency (EMA) already provide guidance for the development of gene editing techniques for therapeutic modification of somatic cells.

While six gene therapy products have already been approved by the FDA and EMA since 2016, more than 2000 are in different stages of clinical approval including several gene editing approaches [1, 58, 84]. Considering the enormous development costs and high prices of current gene therapy products, socio-economic conflicts can be foreseen. While ex vivo gene editing of immune cells might be commercially exploited as cancer treatment, reimbursement for gene editing approaches focusing on rare diseases with individual mutations might become more challenging. Thus, reduction of costs for development and production of gene therapy products is necessary to allow individualized gene editing therapies for rare genetic cardiovascular diseases in the future.

In conclusion, within a few years, gene editing developed from a scientific concept into everyday research reality which now enters the clinic. This development is occurring with breathtaking speed, also owing to the progress in shuttle development, sequencing technology, and bioinformatics. Whereas gene editing just revolutionized basic science, it will soon revolutionize medicine. This will open up treatment avenues for fatal diseases, and cures for many inherent diseases.

## Data Availability

Does not apply.

## Code availability

Does not apply.
